# Positive predictive value of International Classification of Diseases, 10^th^ revision, diagnosis codes for cardiogenic, hypovolemic, and septic shock in the Danish National Patient Registry

**DOI:** 10.1186/s12874-015-0013-2

**Published:** 2015-03-20

**Authors:** Marie Dam Lauridsen, Henrik Gammelager, Morten Schmidt, Henrik Nielsen, Christian Fynbo Christiansen

**Affiliations:** Department of Clinical Epidemiology, Aarhus University Hospital, Olof Palmes Allé 43-45, 8200 Aarhus, Denmark; Department of Anaesthesiology and Intensive Care Medicine, Aarhus University Hospital, Aarhus, Denmark

**Keywords:** Cardiogenic shock, Hypovolemic shock, ICD-10, Inotropic therapy, Positive predictive value, Septic shock, Shock, Validity, Vasopressor therapy

## Abstract

**Background:**

Large registries are important data sources in epidemiological studies of shock, if these registries are valid. Therefore, we examined the positive predictive value (PPV) of diagnosis codes for shock, the procedure codes for inotropic/vasopressor therapy among patients with a diagnosis of shock, and the combination of a shock diagnosis and a code for inotropic/vasopressor therapy in the Danish National Patient Registry (DNPR).

**Methods:**

We randomly selected 190 inpatients with an International Classification of Diseases, 10^th^ revision (ICD-10) diagnosis of shock at Aarhus University Hospital from 2005–2012 using the DNPR; 50 patients were diagnosed with cardiogenic shock, 40 patients with hypovolemic shock, and 100 patients with septic shock. We used medical charts as the reference standard and calculated the PPV with 95% confidence intervals (CI) for overall shock and for each type of shock separately. We also examined the PPV for inotropic/vasopressor therapy and the PPV for shock when a concurrent code for inotropic/vasopressor therapy was also registered.

**Results:**

The PPV was 86.1% (95% CI: 79.7–91.1) for shock overall, 93.5% (95% CI: 82.1–98.6) for cardiogenic shock, 70.6% (95% CI: 52.5–84.9) for hypovolemic shock, and 69.2% (95% CI: 57.7–79.2) for septic shock. The PPV of use of inotropes/vasopressors among shock patients was 88.9% (95% CI: 79.3–95.1). When both a shock code and a procedure code for inotropic/vasopressor therapy were used, the PPV for shock overall was 93.1% (95% CI: 84.5–97.7). ICD-10 codes for subtypes of shock and simultaneously registered use of inotropes/vasopressors provided PPVs of 96.0% (95% CI: 79.6–99.9) for cardiogenic shock, 69.2% (95% CI: 38.6–90.9) for hypovolemic shock, and 82.4% (95% CI: 65.5–93.2) for septic shock.

**Conclusions:**

Overall, we found a moderately high PPV for shock in the DNPR. The PPV was highest for cardiogenic shock but lower for hypovolemic and septic shock. Combination diagnoses of shock with codes for inotropic/vasopressor therapy further increased the PPV of shock overall, and for cardiogenic and septic shock diagnoses.

**Electronic supplementary material:**

The online version of this article (doi:10.1186/s12874-015-0013-2) contains supplementary material, which is available to authorized users.

## Background

Shock is a medical emergency in which organs and tissues of the body are not receiving adequate flow of blood, resulting in decreased oxygen delivery for cell metabolism [1]. Shock is defined as sustained systolic blood pressure below 90 mmHg, or a drop of systolic blood pressure of more than 40 mmHg in patients with hypertension [1]. Moreover, cutaneous, renal, and neurological symptoms are often observed as clinical manifestations of shock, and increased serum lactate is often a biochemical sign of abnormal oxygen metabolism in patients with shock [1]. If left untreated, shock can lead to severe organ damage and death [2,3]. Shock is typically categorized as cardiogenic, hypovolemic, obstructive, or distributive shock. The most common cause of distributive shock is septic shock [4].

Large registries enable efficient and low cost research [5]. However, registry data must be of high validity for use in epidemiologic research.

The positive predictive value (PPV) for shock among patients with myocardial infarction in two Canadian hospital discharge registries has been found to be 75.0% (95% confidence interval [CI]: 19.4–99.4) [6] and 78.8% (95% CI: 67.0–87.9) [6,7]. In addition, the PPV for severe sepsis, including septic shock, is reported to be 70.7% (95% CI: 51.1–90.4) [8]. No previous study has examined the validity of cardiogenic, hypovolemic, or septic shock diagnoses in the Danish National Patient Registry (DNPR). Therefore, we examined the PPV of (1) diagnosis codes for cardiogenic shock, hypovolemic shock, and septic shock in the DNPR overall and for each code separately, (2) treatment codes for inotropic/vasopressor therapy in the DNPR among patients diagnosed with shock, and (3) shock diagnoses when an in-hospital treatment with inotropes/vasopressors is also registered.

## Methods

### Design and setting

We conducted this cross-sectional validation study using data from medical registries in Denmark. The Danish National Health Service provides tax-supported health care to all Danish residents, including universal and free access to general practitioners and hospitals in Denmark [9]. We used the unique 10-digit Civil Person Registration number, assigned to all inhabitants at birth or upon immigration, to identify medical charts [10].

### Identification of patients with shock in the DNPR

We used the DNPR to identify a random sample of 190 patients admitted with cardiogenic, hypovolemic, or septic shock to Aarhus University Hospital from 1 January 2005 through 31 December 2012. We used both primary and secondary inpatient diagnosis codes to identify the sample of shock patients. Data on all somatic hospital admissions have been routinely registered in the DNPR since 1977, and all outpatient and emergency contacts have been included since 1995 [11]. Each admission is registered by one primary diagnosis code and up to 19 secondary diagnosis codes classified according to the International Classification of Diseases, 8^th^ revision (ICD-8) until the end of 1993, and according to the 10^th^ revision (ICD-10) thereafter [11]. Procedures of inotropic/vasopressor therapy have been registered routinely since 2005 [12].

We chose to include a minimum of 40 cases for each shock type to allow sufficient precision of the calculated PPV, and because of the maximum available number of coded cases in the study period for all shock types. With this number of cases, a PPV of 90% for shock subtypes would result in a 95% confidence interval (CI) from 76% to 97%, which we considered acceptable. The total number of patients (190) included in the study and the distribution of patients in the three subgroups of shock were based on the distribution of the three shock subtypes registered in the DNPR in the study period. We randomly included patients of the three shock subtypes registered in the DNPR in the study period until at least 40 patients in each subgroup of shock were reached. Data on treatment with inotropes/vasopressors were obtained by in-hospital treatment codes during hospitalization from the DNPR (codes provided in Additional file 1: Table e1).

We obtained data on age and sex from the Danish Civil Registration System [10].

To assess the level of comorbidities, we used the Charlson Comorbidity Index (CCI) [13]. The CCI is a validated scoring system that has been adapted for use with hospital discharge data [14]. The scoring system assigns a point score from 1 to 6 to a number of diseases depending on their relation to mortality, defined during a one-year period in which the scoring was made [13]. The scoring system generated three levels of comorbidity: 0 (low), 1–2 (moderate), and ≥ 3 (high). For each patient, we used a fixed period of 5 years prior to hospital admission with shock, and included any inpatient or outpatient hospital visits with a diagnosis of the diseases, including both prevalent and incident diseases. ICD-10 codes are provided in Additional file 1: Table e2.

### Medical chart review

One author (MDL) obtained and reviewed all available medical charts. Each chart was reviewed, including all notes from date of hospital admission with shock to the date of hospital discharge. This approach was chosen to confirm shock diagnosis and type of shock by predefined diagnostic criteria (Table 1), and to confirm treatment with inotropes/vasopressors.

**Table 1:** **Diagnostic criteria used to validate shock diagnoses.**

**Shock type**	**Diagnostic criteria**
Shock overall	Diagnosis of shock is confirmed if sustained shock^a^ can be confirmed by medical chart review by at least one of the following [1,15]:^b^
	1. Systolic blood pressure < 90 mmHg.
	2. Mean arterial pressure < 70 mmHg.
	3. Reduction in systolic blood pressure > 40 mmHg.
	4. Preserved systolic blood pressure achieved through inotropic/vasopressor therapy.
Cardiogenic shock	Diagnosis of cardiogenic shock is confirmed if medical chart review confirms these two criteria [4,16]:
	1. Sustained shock (as defined in shock overall) [1,15], and
	2. Two or more of the following criteria were confirmed:
	a. Cardiac index < 2.2 (L/min)/m^2^,
	b. pulmonary capillary wedge pressure > 18 mmHg,
	c. tachycardia (>90 beats per minute),
	d. pale, cold, clammy, or cyanotic skin,
	e. signs of oliguria, or
	f. confusion, disorientation, or loss of conscience.
Hypovolemic shock	Diagnosis of hypovolemic shock is confirmed if medical chart review confirms these two criteria:
	1. Sustained shock (as defined in shock overall) [1,15]
	2. Evidence or suspicion of shock due to (one or more of the following) [4]:
	a. Loss of red blood cell mass and plasma from hemorrhage.
	b. Loss of plasma volume alone due to extravascular fluid sequestration.
	c. Gastrointestinal, urinary, and insensible losses.
Septic shock	Diagnosis of septic shock is confirmed if medical chart review confirms these three criteria [1,15,17]:
	1. Sustained shock (as defined in shock overall) [1,15]
	2. Systemic inflammatory response syndrome (SIRS) must be diagnosed by identifying at least two or more of the following:
	a. Tachypnea: high respiratory rate) > 20 breaths per minute, or arterial blood gas, with PCO2 less than 4.3 kPa signifying hyperventilation.
	b. White blood cell count either significantly low < 4000 cells/mm^3^, elevated > 12000 cells/mm^3^, or >10 immature cells.
	c. Heart rate > 90 beats per minute.
	d. Temperature: Fever > 38.3°C (100.4°F) or hypothermia < 36.0°C (96.8°F).
	3. Sepsis and not an alternative form cause of SIRS. Sepsis requires evidence or suspicion of infection, which may include:
	a. Positive blood culture/blood culture taken as suspicious of infection,
	b. signs of pneumonia on chest x-ray, or
	c. other radiologic or laboratory evidence of infection.

^a^Sustained shock defined as shock > 30 minutes despite adequate fluid resuscitation.

^b^Blood pressure used as a surrogate for decreased blood flow.

*Abbreviation:* *SIRS* Systemic inflammatory response syndrome.

### Statistical analysis

We tabulated patient characteristics by shock type, including 1) median age and interquartile range, 2) gender and level of comorbidity as counts and percentages.

We used the STATA command *diagt* (diagnostic test) to estimate the PPV as the proportion of registered diagnoses confirmed by the predefined criteria during medical chart review. Ninety-five percent CIs are given as the exact binomial CI. The calculations were done under the assumption that all the missing medical charts did not differ from the charts found in the archives with respect to the proportion of confirmed shock cases. To ensure that no substantial differences existed between patients with available medical charts and patients with missing medical charts, we (1) compared the two groups of patients with regard to gender, age, and level of comorbidity, (2) examined whether age group, gender, and level of comorbidity were predictors of misclassification using a logistic regression model with verified shock as the outcome, and (3) conservatively estimated the PPV for shock and subtypes of shock by including missing medical charts as non-confirmed shock.

We calculated the PPV for use of inotropes/vasopressors among patients having a diagnosis code of shock. Patients that have a confirmed treatment with inotropes/vasopressors in the medical chart were used as the numerator, and patients with a procedure code of inotropic/vasopressor therapy defined the denominator.

To examine whether the PPVs for shock overall and shock subtypes could be improved using a concurrent procedure code of inotropic/vasopressor therapy, we estimated the PPV as the proportion of confirmed shock cases among patients registered with both a shock diagnosis and an inotropic/vasopressor code. For all statistical analyses, we used STATA statistical software version 13.1 (StataCorp LP, Texas).

### Research ethics and informed consent

This validation study was non-experimental and solely used existing data; thus, ethical approval was not needed. The study was approved by the Danish Data Protection Agency (record no. 2006-53-1346).

## Results

### Patient characteristics

**Figure 1:**
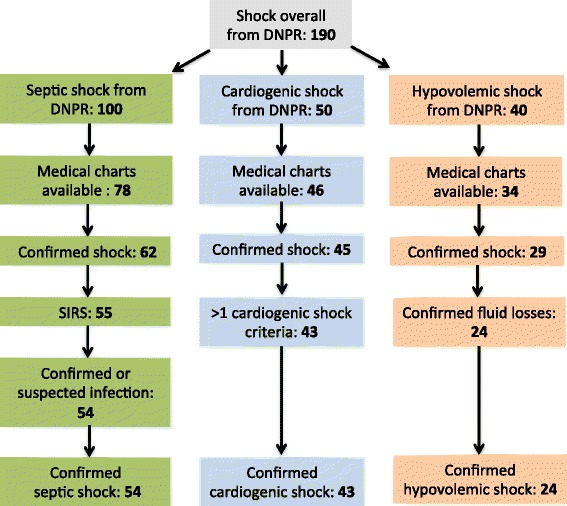
**Flowchart of study population.** Overview of patient selection from the DNPR, available medical charts, and confirmed diagnostic criteria for subtypes of shock.

Among the medical charts reviewed, 46 of 50 (92%) were available for cardiogenic shock validation, 78 of 100 (78%) were available for septic shock validation, and 34 of 40 (85%) were available for hypovolemic shock validation. A flowchart showing the selection process is provided in Figure 1.

Patients with missing charts were slightly younger, and the majority were men (Additional file 1: Table e3). The level of comorbidity did not differ substantially between patients with and without an available medical chart (Additional file 1: Table e3).

The median age at hospital admission among patients with a code of shock was 71 years (interquartile range: 61–80), and the distribution between males and females was equal (Table 2). Patients with cardiogenic shock had an overall lower level of comorbidity compared with patients with hypovolemic and septic shock (Table 2).

**Table 2:** **Age, gender, and comorbidity among patients with an available medical chart**

	**Shock type**			
**Covariates**	**Shock overall 158 (100)** ^**a**^	**Cardiogenic shock 46 (100)** ^**a**^	**Hypovolemic shock 34 (100)** ^**a**^	**Septic shock 78 (100)** ^**a**^
**Demographics**				
Age, median, (IQR)	71 (61–80)	70 (59–76)	69 (60–82)	73 (63–81)
Male gender	77 (49)	25 (54)	14 (41)	38 (49)
**CCI** ^**b**^ **score**				
Low	52 (33)	25 (54)	10 (29)	17 (22)
Moderate	57 (36)	14 (30)	10 (29)	33 (42)
High	49 (31)	7 (15)	14 (41)	28 (36)

^a^Values expressed as count (percentage) unless otherwise indicated.

^b^Three levels of comorbidity were defined based on Charlson Comorbidity Index scores of 0 (low), 1–2 (moderate), and ≥3 (high).

*Abbreviations:* *CCI* Charlson comorbidity index, *IQR* Interquartile range.

### Positive predictive value for shock and subtypes of shock

The PPV for shock overall was 86.1% (95% CI: 79.7–91.1) (Table 3). The PPVs were 93.5% (95% CI: 82.1–98.6) for cardiogenic shock, 70.6% (95% CI: 52.5–84.9) for hypovolemic shock, and 69.2 % (95% CI: 73.3–94.2) for septic shock (Table 3). Patients who did not fulfill the diagnostic criteria were equally distributed in groups of patients with unconfirmed shock and patients not fulfilling the specific shock subtype criteria (Figure 1).

**Table 3:** **Positive predictive values in the Danish National Patient Registry with shock diagnoses, inotropic/vasopressor therapy among patients with shock, and shock from the combination of diagnoses and inotropic/vasopressor therapy**

	**Shock** ^**a**^	**No shock**	**Total number** ^**b**^	**Positive predictive value, % (95% CI)**
**Diagnosis code** ^**c**^				
Shock	136	22	158	86.1 (79.7–91.1)
Cardiogenic shock	43	3	46	93.5 (82.1–98.6)
Hypovolemic shock	24	10	34	70.6 (52.5–84.9)
Septic shock	54	24	78	69.2 (57.7–79.2)
**Procedure code** ^**c**^				
Inotropes/vasopressors	67	5	72	93.1 (84.5–97.7)
**Diagnosis code** ^**c**^ **+ inotropic/vasopressor code** ^**c**^				
Cardiogenic shock	24	1	25	96.0 (79.6–99.9)
Hypovolemic shock	9	4	13	69.2 (38.6–90.9)
Septic shock	28	6	34	82.4 (65.5–93.2)

^a^Shock defined as a confirmed overall shock or subtype of shock by medical chart review.

^b^All missing medical charts are excluded.

^c^DNPR codes: shock overall (R570, R571, R572, A41.9A), cardiogenic shock (R570), hypovolemic shock (R571), septic shock (R572, A41.9A), and inotropes/vasopressors (BFHC92, BFHC93 (excluding BFHC93E-BFHC93H), BFHC95.

*Abbreviation*: *CI* Confidence interval.

When we included missing medical charts as unconfirmed shock in the calculations, the PPV was 71.6% (95% CI: 65.2–78.0) for shock overall, 86.0% (95% CI: 73.3–94.2) for cardiogenic shock, 60.0% (95% CI: 43.3–75.1) for hypovolemic shock, and 54.0% (95% CI: 43.7–64.0) for septic shock (Additional file 1: Table e4).

Male gender, age less than 60 years, and a high level of comorbidities may be predictors of misclassification, although estimates were imprecise (Additional file 1: Table e5).

### Predictive values for treatment with inotropes/vasopressors

We found that 72 of the 158 patients diagnosed with shock and with a medical chart available for review had a code for inotropes/vasopressors. Treatment with inotropes/vasopressors was confirmed in the medical chart in 64 of the 72 cases, providing a PPV of 88.9% (95% CI: 79.3–95.1).

### Predictive values for combined shock and inotrope/vasopressor codes

The PPV for the shock diagnosis code in combination with inotropes/vasopressors was 93.1% (95% CI: 84.5–97.7) (Table 3). The PPV for shock subtypes combined with inotropic/vasopressor therapy was 96.0% (95% CI: 89.6–99.9) for cardiogenic shock, 69.2% (95% CI: 38.6–90.9) for hypovolemic shock, and 82.4% (95% CI: 65.5–93.2) for septic shock (Table 3).

## Discussion

We found a moderately high PPV for shock diagnoses in the DNPR. For subtypes of shock, cardiogenic shock diagnosis had the highest PPV, whereas more patients diagnosed with hypovolemic and septic shock did not fulfill the diagnostic criteria for these two types of shock.

Procedure codes for treatment with inotropes/vasopressors had also a high PPV. The PPVs for shock were improved when the diagnosis code for shock overall and cardiogenic or septic shock were combined with the procedure code for inotropes/vasopressors. However, the PPV was unchanged for hypovolemic shock when combined with procedure codes for inotropes/vasopressors.

### Existing studies

Our finding for the PPV for cardiogenic shock is somewhat higher than results from hospital discharge registries from Canada [6,7]. Lawrence et al. found a PPV of 75.0% (95% CI: 19.4–99.4) for cardiogenic shock among patients with acute myocardial infarction in a hospital discharge registry in Alberta, Canada [6]. However, the study was of limited size, with only four patients registered with ICD-10 data for shock [6]. Another Canadian study of 66 patients diagnosed with an International Classification of Diseases, 9^th^ revision (ICD-9) code for cardiogenic shock in Quebec’s hospital discharge registry reported a PPV of 78.8% (95% CI: 67.0–87.9) [7]. Similar to our finding for septic shock, a study from the University of Michigan Health Systems examined the PPV for ICD-9 codes for severe sepsis, including septic shock, among 111 patients and found a PPV of 70.7% (95% CI: 51.1–90.4) [8].

### Strengths and limitations

Because we reviewed medical chart data from patients with a registered ICD-10 diagnosis code of shock, we could only estimate the PPV and not other measures of validity including negative predictive value, sensitivity, and specificity. Other studies found the sensitivity for myocardial infarction-related shock code to be 60% [6,7] and specificity to be 99% [6,7]. Because we aimed to validate the PPV and not sensitivity of the shock diagnoses, it was not necessary to include a control group.

The medical chart review was not blinded, which could have influenced the data collection. However, strict clinical criteria in congruity with internationally-approved diagnostic criteria [1,15,16] for each shock subtype were defined before the beginning of the data collection; thus, the potential of subjective influence from the data collector was limited. Still, it is a potential limitation that only one author reviewed the data from the medical charts.

Because clinical cases do not always fulfill all diagnostic criteria, some cases are coded with the most suitable code for the course of the disease. Register-based research relies on correct coding procedures, and the PPVs for septic shock and hypovolemic shock reveal some degree of coding inaccuracy. It should also be noted that the criteria for defining shock subtypes may not be mutually exclusive, which may have influenced the PPVs for shock.

Medical charts were not available for all patients in our sample. No substantial differences in the available comorbidity level were found between the two groups of patients, which is why the assumption of no difference between patients with missing charts and those with charts was not disproved.

This study was conducted at Aarhus University Hospital, and all medical charts were randomly selected from the DNPR, including 15 different departments in the hospital. Coding practices between hospital settings might vary. However, the diagnostic criteria for shock subtypes follow national guidelines, which likely make our results representative to other hospitals in Denmark.

No previous study has examined the PPV of shock codes registered in the DNPR, despite the fact that the DNPR may be an important tool for research and quality monitoring of shock. We found fairly high PPVs for shock overall, and for subtypes of shock, which makes these ICD-10 codes usable in studies of risk and prognosis based on data from the DNPR. However, the potential impact of misclassification of shock should be considered when conducting such studies.

## Conclusions

We found a moderately high PPV for shock overall in the DNPR using the medical chart as the reference standard. PPV was highest for cardiogenic shock and lower for hypovolemic and septic shock. A feasible approach to increase the PPV of shock overall and of septic shock is to combine the diagnosis with the procedure codes for treatment with inotropes/vasopressors. In summary, the DNPR is a valuable tool for epidemiological research of shock and subtypes of shock, especially cardiogenic shock.

## Additional file

Additional file 1:
**Content: Tables e1-5, electronic file name.**

## Abbreviations

CCI: Charlson comorbidity index
CI: Confidence interval
DNPR: The Danish National Patient Registry
ICD-10: International Classification of Diseases, 10^th^ revision
ICD-8: International Classification of Diseases, 8^th^ revision
ICD-9: International Classification of Diseases, 9^th^ revision
PPV: Positive predictive value
