# Supplementary material for: Positive predictive value of International Classification of Diseases, 10th revision, diagnosis codes for cardiogenic, hypovolemic, and septic shock in the Danish National Patient Registry
Source: BMC Med Res Methodol. 2015 Mar 20;15:23. doi: 10.1186/s12874-015-0013-2 (PMC4373092; doi:10.1186/s12874-015-0013-2)
Supplement: Additional file 1: — Content: Tables e1-5, electronic file name. [file 12874_2015_13_MOESM1_ESM.pdf]

Additional file (table e1-e5):

Table e1: Codes used to retrieve data from the Danish National Patient Registry [a]

Description	Codes
Treatment with inotropes or vasopressors	Procedure codes: BFHC92, BFHC93, BFHC95 (excluding BFHC93E-BFHC93H)
Cardiogenic shock	ICD-10: R570
Hypovolemic shock	ICD-10: R571
Septic shock	ICD-10: R572, A41.9A

Abbreviations: ICD-10: International Classification of Diseases, 10th revision

[a] Both primary and secondary in-patient diagnosis codes used.

Table e2: Charlson Comorbidity Index score

**Charlson comorbidity category**		**ICD-10**	**Charlson Score**
1	Myocardial infarction	I21; I22; I23	1
2	Congestive heart failure	I50; I11.0; I13.0; I13.2	1
3	Peripheral vascular disease	I70; I71; I72; I73; I74; I77	1
4	Cerebrovascular disease	I60-I69; G45; G46	1
5	Dementia	F00-F03; F05.1; G30	1
6	Chronic pulmonary disease	J40-J47; J60-J67; J68.4; J70.1; J70.3; J84.1; J92.0; J96.1; J98.2; J98.3	1
7	Connective tissue disease	M05; M06; M08; M09; M30; M31; M32; M33; M34; M35; M36; D86	1
8	Ulcer disease	K22.1; K25-K28	1
9	Mild liver disease	B18; K70.0-K70.3; K70.9; K71; K73; K74; K76.0	1
10	Diabetes type1	E10.0, E10.1; E10.9	1
	Diabetes type2	E11.0; E11.1; E11.9	
11	Hemiplegia	G81; G82	2
12	Moderate to severe renal disease	I12; I13; N00-N05; N07; N11; N14; N17-N19; Q61	2
13	Diabetes with end organ damage		2
	type1	E10.2-E10.8	
	type2	E11.2-E11.8	
14	Any tumor	C00-C75	2
15	Leukemia	C91-C95	2
16	Lymphoma	C81-C85; C88; C90; C96	2
17	Moderate to severe liver disease	B15.0; B16.0; B16.2; B19.0; K70.4; K72; K76.6; I85	3
18	Metastatic solid tumor	C76-C80	6
19	AIDS	B21-B24	6

Abbreviations: AIDS: Acquired immune deficiency syndrome, ICD-10: International classification of diseases, 10th revision

Table e3: Gender, age and Charlson Comorbidity Index score among patients with a missing medical file.

Co-variates	Available medical chart<br>Shock type<br>Cardiogenic<br>46 (100)[a]	 <br>Hypovolemic<br>34 (100)	 <br>Septic<br>78 (100)	Medical files missing<br>Shock type<br>Cardiogenic<br>4 (100)	 <br>Hypovolemic<br>6 (100)	 <br>Septic<br>22 (100)
**Demographics**						
Age, median, (IQR)	70.0 (59-76)	68.5 (60-82)	72.5 (63-81)	68.5 (65-75)	65.5 (36-79)	64.5 (60-78)
Gender (male)	25 (54.4)	14 (41.2)	38 (48.7)	4 (100)	3 (50.0)	13 (59.1)
**CCI score[b]**						
Low	25 (54.4)	10 (29.4)	17 (21.8)	3 (75.0)	5 (83.3)	5 (22.7)
Moderate	14 (30.4)	10 (29.4)	33 (42.3)	0 (0)	0 (0)	11 (50.0)
High	7 (15.2)	14 (41.2)	28 (35.9)	1 (25.00)	1 (16.7)	6 (27.3)

Abbreviations: CCI: Charlson comorbidity index, IQR: Inter quartile range

[a] Values expressed as count (percentage) unless otherwise indicated

[b] Three levels of comorbidity were defined based on Charlson Comorbidity Index scores of 0 (low), 1–2 (moderate), and ≥3 (high).

Table e4: Positive predictive values for shock overall and subtypes of shock (cardiogenic, hypovolemic and septic shock) including missing files.

Diagnosis code	Medical files		Total number of cases	Positive predictive value (%) (95% CI)
	Shock	No shock		
Shock	136	54	190	71.6 (65.2-78.0)
Cardiogenic shock	43	7	50	86.0 (73.3-94.2)
Hypovolemic shock	24	16	40	60.0 (43.3-75.1)
Septic shock	54	46	100	54.0 (43.7-64.0)

Table e5: Age, gender and Charlson Comorbidity Index score as predictors for misclassification of shock overall.

	Shock misclassification odds ratio (95% CI)
Age group	
<60	1 (reference)
60-69	0.61 (0.18-2.06)
70-79	0.32 (0.08-1.20)
80 ≤	0.40 (0.10-1.52)
Gender	
Female	1 (reference)
Male	1.44 (0.56-3.69)
CCI score[a]	
Low	1 (reference)
Moderate	0.92 (0.27-3.08)
High	1.64 (0.54-5.04)

Abbreviations: CCI: Charlson comorbidity index

[a] Three levels of comorbidity were defined based on Charlson Comorbidity Index scores of 0 (low), 1–2 (moderate), and ≥3 (high).
